# The Chemo-Sensitizing Effect of Doxorubicin of Apple Extract-Enriched Triterpenic Complex on Human Colon Adenocarcinoma and Human Glioblastoma Cell Lines

**DOI:** 10.3390/pharmaceutics14122593

**Published:** 2022-11-24

**Authors:** Aurita Braciuliene, Valdimaras Janulis, Vilma Petrikaite

**Affiliations:** 1 Department of Pharmacognosy, Lithuanian University of Health Sciences, Sukileliu Av. 13, LT-50162 Kaunas, Lithuania; 2 Laboratory of Drug Targets Histopathology, Institute of Cardiology, Lithuanian University of Health Sciences, Sukileliu Av. 13, LT-50162 Kaunas, Lithuania

**Keywords:** anticancer, apple, DOX, triterpene, HT-29, U-87

## Abstract

Cancer cells’ resistance to anticancer drugs represents a major clinical problem and the most important failure of treatment. Combination chemotherapy is more effective than monotherapy due to additive or synergistic effects. The aim of our research was to assess the effects of the combinations of apple extract’s triterpenic compounds, individual triterpenic acids, and doxorubicin (DOX) on human colon adenocarcinoma (HT-29) and human glioblastoma (U-87) cell lines in 2D and 3D cultures. The effect of the combination of apple extracts, the triterpenic standards, and DOX against HT-29 and U-87 cell viability was tested by the MTT and spheroid growth assays. Cell line HT-29 was more sensitive to DOX when incubated with all tested apple extracts than DOX alone. Cell line HT-29 was the most strongly sensitive to DOX when it was treated with 5 µM oleanolic acid (change of EC_50_ = −64.6% ± 4.4%) and with 5 µM ursolic acid (change of EC_50_ = −61.9% ± 8.8%) in 2D culture. Meanwhile, cell line U-87 was the most strongly sensitive to DOX when treated with 2 µM betulinic acid (change of EC_50_ = −45.1% ± 4.5%) in 2D culture. The combination of apple extract (E3) and DOX reduced the viability of HT-29 spheroids the most (spheroid viability reduced from −19.9% to −10.9%, compared to spheroids treated with DOX alone). Our study in 2D and 3D cultures showed that combining apple extract’s triterpenic complexes or individual triterpenic acids with DOX may sensitize chemotherapeutic drugs and increase the cytotoxicity effects in HT-29 and U-87 cell lines.

## 1. Introduction

Cancer is one of the prevailing reasons for morbidity and mortality worldwide. Based on Global Cancer Statistics data, in 2020, 18.1 million new cancer cases were established, with colon cancer representing 10.7% of all cases, and brain and central nervous system cancers 1.7% of all cases [1]. Colon cancer is the third most common and the second most deadly cancer globally [1]. Meanwhile, glioblastoma is the deadliest form of brain tumor, encompassing more than 50% of primary brain cancers [2,3]. A major problem in clinical chemotherapy is that cancer cells can become resistant to cytotoxic drugs [4,5]. The multidrug resistance (MDR) toward chemotherapeutic agents is related to cellular changes, such as reduced drug accumulation, enhanced drug efflux and detoxification potency, and subcellular reallocation. One of the most prevalent mechanisms of cancer cell resistance includes the efflux of chemotherapeutic agent molecules out of the cells [5]. According to scientific literature, ATP-binding cassette family transporters, especially P-glycoprotein, the most characterized protein of this family, are responsible for the accelerated removal of drug molecules from cells [6,7]. The next important mechanism for cancer cells’ resistance to drugs is modifying drug targets by altering the expression of genes and proteins related to tumorigenesis and increasing the production of anti-apoptotic proteins such as Bcl-2 and Bcl-Xl [4].

Doxorubicin is one of the most effective chemotherapeutic agents used routinely in the treatment of colon cancer and other solid tumors [7,8,9]. The chemotherapeutic effect of doxorubicin is achieved via two mechanisms. First, the doxorubicin molecule intercalates into the DNA double helix, consequently inhibiting topoisomerase-II, and second, this agent promotes reactive oxygen species formation, therefore damaging DNA, proteins, and cell membranes [10]. However, the treatment of doxorubicin has challenges. Doxorubicin’s therapeutic utility is narrow and the cure with this anticancer drug is related to cardiac adverse effects, which can lead to a heart attack [11]. Doxorubicin is a P-glycoprotein substrate, so most cancer cells are resistant to this drug [12]. P-glycoprotein transports neutral or positively charged lipophilic agents and can efflux a broad spectrum of clinically necessary chemotherapeutic agents, such as doxorubicin, vincristine, and paclitaxel [13,14].

Chemotherapy, regardless of its many adverse effects, is still the most acceptable way of curing cancer [15]. Cancer cells’ resistance to chemotherapeutic drugs represents a major clinical problem and the most important failure of treatment; therefore, from the current point of view, there is a need to search for modern, safe, and more effective methods of chemotherapy [7]. Particularly, many studies have demonstrated that some natural products in combination with chemotherapeutic agents can have chemoprotective or synergistic effects in terms of reducing cancer chemotherapy-associated side effects and enhancing the therapeutic efficacy [16]. Some authors described that combination chemotherapy is more effective than monotherapy due to additive or synergistic effects [17,18,19]. Combining natural agents with the chemotherapeutic drug might suggest a beneficial approach to cancer treatment [20]. A diet rich in biologically active compounds containing foods could be used as the first line of protection, or as a component of traditional chemotherapy [21]. A previous study has shown that more use of fruits and vegetables can preclude 20% of the incidence of cancer and about 200,000 cancer-involved deaths annually [22]. On a global scale, apples are one the most consumed fruits in the world and are not only a relevant source of sugars, organic acids, fiber, vitamins, and macro- and microelements, but also possess various phytochemicals, such as triterpenic and phenolic compounds which are characterized by various biological and pharmacological activities [23]. Triterpenic compounds are secondary metabolites, have versatile biological effects, and may act at the cellular level. Primarily, triterpenic compounds can suppress proliferation and provoke the apoptosis of cancer cells, diminish their invasive ability, and have anti-inflammatory and cytoprotective activity on normal cells [24].

Combination administration applying standard anticancer drugs and natural biological compounds extracted from the plant, or entire plant extracts, may be one of the most valuable strategies to defeat drug resistance. According to the scientific literature, most studies assess the effect of the combination of phenolic compounds and chemotherapy drugs on cancer cells. Shakibaei et al. showed that curcumin enhanced the effect of 5-fluorouracil on the colorectal HCT116 and HCT116 + ch3 cancer cell lines [25]. Staedler et al. determined the potency of quercetin to enhance the effects of doxorubicin in human breast cancer MDA-MB-231 and MCF-7 cell lines [26]. Bracci et al. demonstrated that the combination of quercetin and doxorubicin inhibited colon CT26 cancer cell growth and diminished cardiotoxicity in vivo [27]. However, we found only fragmentary data on combining chemotherapeutics and triterpenic compounds, one of the most widespread groups of biologically active compounds with a wide spectrum of anticancer effects. In our study, we combined the chemotherapeutic agent doxorubicin with apple extract’s triterpenic compounds complex and individual triterpenic acids, and we aimed to evaluate the effect of these combinations on human colon adenocarcinoma HT-29 and human glioblastoma U-87 2D and 3D cell cultures. In the scientific literature, until now we did not find any studies evaluating the effects of combining apple extracts and the anticancer drug doxorubicin on these cell lines in 3D cultures. Data from our study open new perspectives for improving the chemotherapeutic success of MDR cancer cures. This strategy combining apple extracts with doxorubicin is valuable for enhancing the effectiveness of chemotherapy, improving cancer cells’ reaction to the drugs, reducing the toxic doses of drugs, and consequently, decreasing undesirable side effects.

The aim of our research was to assess the effects of the combinations of apple extracts, triterpenic acids, and doxorubicin on HT-29 and U-87 cell lines in 2D and 3D cultures.

## 2. Materials and Methods

### 2.1. Plant Materials

This research involved the apple cultivars ‘Auksis’, ‘Kostele’, ‘Ligol’, and ‘Rubin’, which were cultivated in the orchard of the Institute of Horticulture, Lithuanian Research Centre for Agriculture and Forestry, Babtai, Lithuania (55°600 N, 23°480 E). The apple trees were cultivated in 2020–2021.

### 2.2. Cell Cultures

The human colon adenocarcinoma cell line HT-29 and human glioblastoma cell line U-87 were purchased from the American Type Culture Collection (ATCC, Manassas, VA, USA). Human fibroblasts were provided by prof. Helder A. Santos (University of Helsinki, Helsinki, Finland). Cells were cultured in DMEM Glutamax medium supplemented with 10% fetal bovine serum (FBS), 10,000 U/mL penicillin, and 10 mg/mL streptomycin solution, in a humidified atmosphere containing 5% CO_2_ at 37 °C. Cell cultures were grown to 70% confluency, then trypsinized with 0.125% TrypLE Express solution before passage, and used until passage 20.

### 2.3. Chemicals

All solvents, reagents, and standards used were of analytical grade. Standards used in HPLC analysis: ursolic acid (UA), oleanolic acid (OA), corosolic acid (CA), and betulinic acid (BA), were purchased from Sigma-Aldrich GmbH (Buchs, Switzerland). Solvents used in the study: 99.9% acetonitrile and 99.9% acetone, were acquired from Sigma-Aldrich GmbH (Buchs, Switzerland), and 99.5% dimethyl sulfoxide (DMSO) was purchased from Scharlab S.L. (Barcelona, Spain). In this study, we used deionized water prepared by a Milli-Q (Millipore, Bedford, MA, USA) water purification system. Doxorubicin (DOX) hydrochloride was bought from Abcam (Cambridge, UK), and 3-(4,5-dimethylthiazol-2-yl)-2,5-diphenyl tetrazolium bromide (97.0%, MTT) was purchased from Buchs GmbH, Switzerland. The plasticware for cell cultures was obtained from Techno Plastic Products (Trasadingen, Switzerland), Corning (Corning, NY, USA), and Thermo Fisher Scientific (Waltham, MA, USA). Dulbecco’s modified Eagle high-glucose medium (DMEM Glutamax), TrypLETM Express reagent, fetal bovine serum (FBS), penicillin/streptomycin solution (100×), and phosphate-buffered saline (PBS) were obtained from Gibco (Thermo Fisher Scientific Inc., Waltham, MA, USA). NanoShuttle magnetic nanoparticles were purchased from Nano3D Biosciences Inc. (Houston, TX, USA).

### 2.4. Preparation of Apple Samples and Dry Triterpenic Extracts

Apple samples and dry triterpenic extracts were prepared using the method described by Butkeviciute et al. [28]. In this study, we applied apple extracts abbreviations (E1—extract of apple peel of ‘Ligol’ cultivar, E2—extract of apple peel of ‘Rubin’ cultivar, E3—extract of apple peel of ‘Auksis’ cultivar, and E4—extract of the whole apple of ‘Kostele’ cultivar). The qualitative and quantitative composition of triterpenic compounds of the extracts was described in our previous study, reported by Butkeviciute et al. [28].

### 2.5. Analysis of Triterpenic Compounds

The triterpenic compounds were identified and quantified by the HPLC-PDA method in the same manner reported in previous research by Butkeviciute et al. [29].

### 2.6. Cell Viability

The effect of the combination of apple extracts, the triterpenic standards, and DOX against human colon adenocarcinoma HT-29 and glioblastoma U-87 cell viability was tested by the MTT assay, as reported by Grigalius and Petrikaite [30]. The concentration of apple extracts was 30 µg/mL, ursolic and oleanolic acids were 5 µM, and corosolic and betulinic acids were 2 µM. Doxorubicin 5 × dilutions were performed in the medium: 1 µM, 200 nM, 40 nM, 8 nM, 1.6 nM, and 0.32 nM, and added to the cells in triplicates. The cells not treated with doxorubicin were used as a negative control (their viability corresponded to 1.0), and the positive control was the cells with 5 µM of doxorubicin, which killed all cells (cell viability corresponded to 0). After measuring the absorbance of the obtained MTT solutions, the half-maximal effective concentration (EC_50_) values were calculated using the Hill equation. The EC_50_ value represents the concentration of a compound causing a 50% reduction of cancer cell metabolic activity.

The change of EC_50_ (expressed in %) was established, which indicates how much DOX toxicity changed when the apple extracts and individual triterpenic acids solution were added to the medium. The experiments were repeated three times independently, and the results are presented as the means ± SD.

### 2.7. Spheroid Growth

Spheroids were formed by a magnetic 3D bioprinting method, as described elsewhere [31]. Briefly, the cancer cells were mixed with human fibroblasts (at a ratio of 1:1) to better represent the tumor microenvironment. The cells were then incubated with 20 µL nanoparticles for 8–10 h, then resuspended and seeded into ultra-low attachment 96-well plates at a volume of 100 µL (1000 glioblastoma or colorectal carcinoma cells and 1000 human fibroblasts per well). The plate was placed on a magnetic drive and incubated in a humidified atmosphere containing 5% CO_2_ at 37 °C, until spheroids formed. After 48 h of incubation, the tested compounds and extracts were added. The DOX concentration used in spheroids was 20 nM, and the concentrations of tested material were as follows: 2 µM of triterpenic acids, and 30 µg/mL of extracts. In addition, control groups were included: the spheroids not treated with any substances or only with tested triterpenic acids and extracts (without doxorubicin).

Photos of spheroids were taken every 48 h, simultaneously changing the medium with tested materials. The effect of the combination of apple extract’s triterpenic compounds, individual triterpenic acids, and DOX in 3D glioblastoma and colorectal carcinoma cells was analyzed by measuring the diameter change of spheroids using ImageJ software (National Institutes of Health, Bethesda, MD, USA) and by the change of cell viability in spheroids by the MTT assay. The experiments were repeated three times independently, and the results are presented as the means ± SD.

### 2.8. Data Analysis

The statistical analysis of the study data was performed using Microsoft Office Excel 2013 (Microsoft, Redmond, WA, USA) and SPSS 25.0 (SPSS Inc., Chicago, IL, USA) computer software. All the experiments were performed in at least triplicate independent measurements and the obtained values were reported as mean ± standard deviation. Tukey’s multiple comparison test was used. The differences were held as statistically significant at *p* < 0.05.

## 3. Results and Discussion

### 3.1. Effect of Apple Extracts and Triterpenic Acids on DOX in HT-29 and U-87 Cancer Cells (2D Cultures)

**Figure 1:**
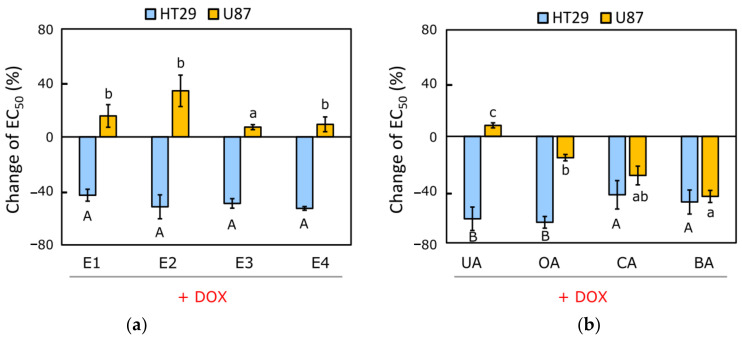
Effect of triterpenic compounds on DOX in human colon adenocarcinoma (HT-29) and human glioblastoma (U-87) cell lines: (**a**) Effect of apple extract′s triterpenic compounds’ complexes on DOX in HT-29 and U-87 cancer cell lines. (**b**) Effect of purified individual triterpenic acids on DOX in HT-29 and U-87 cancer cell lines. Abbreviations: E1—extract of apple peel of ‘Ligol’ cultivar; E2—extract of apple peel of ‘Rubin’ cultivar; E3—extract of apple peel of ‘Auksis’ cultivar; E4—extract of the whole apple of ‘Kostele’ cultivar; UA—ursolic acid; OA—oleanolic acid; CA—corosolic acid; BA—betulinic acid. Different uppercase and lowercase letters indicate statistically significant differences at *p* < 0.05.

Based on the results, the HT-29 cell line was more sensitive to DOX when incubated with all apple extracts than with DOX alone (Figure 1a, Tables S1 and S3). All extracts, E1, E2, E3, and E4, sensitized the cell line HT-29 to DOX in a similar manner (change of EC_50_ varied from −43.2% ± 2.2% to −53.0% ± 1.4%), and there were no statistically significant differences between them. Cell line U-87 became even less sensitive to DOX when treated with all apple extracts (change of EC_50_ varied from 7.2% ± 0.4% to 34.2% ± 1.7%) (Figure 1a).

According to the results, HT-29 and U-87 cells became more sensitive to DOX when treated with ursolic, oleanolic, corosolic, and betulinic acids (Figure 1b, Tables S2 and S3). Experimental triterpenic acids are the most commonly established pentacyclic triterpenes in apple samples [29]. Cell line HT-29 was sensitized to DOX after the treatment with all triterpenic acids (change of EC_50_ varied from −64.6% ± 4.4% to −43.9% ± 10.7%) (Figure 1b). Cell line HT-29 was the most strongly sensitive to DOX when treated with 5 µM oleanolic acid (change of EC_50_ = −64.6% ± 4.4%) and with 5 µM ursolic acid (change of EC_50_ = −61.9% ± 8.8%) (Figure 1b). Cell line HT-29 was less sensitive to DOX when treated with 2 µM corosolic acid (change of EC_50_ = −43.9% ± 10.7%) or with 2 µM betulinic acid (change of EC_50_ = −49.3% ± 9.0%), though the effect was still synergistic (Figure 1b). Cell line U-87 was less sensitive to the DOX and triterpenic acids treatment (oleanolic, corosolic, and betulinic acids) (change of EC_50_ varied from −45.1% ± 4.5% to −16.0% ± 2.4%) (Figure 1b). Cell line U-87 was the most strongly sensitive to DOX when treated with 2 µM betulinic acid (change of EC_50_ = −45.1% ± 4.5%). We found that cell line U-87 was not sensitized to DOX when treated with 5 µM ursolic acid (Figure 1b).

Miscellaneous fruits, including apples, can be held as possible sources of a chemosensitizer [32]. For some time, triterpenic compounds were considered biologically inactive, but research has proven their broad-spectrum pharmacological effects with a low toxicity profile, so the interest in triterpenic compounds as natural-origin, biologically active compounds has rapidly increased [33]. Previous studies have established the multifaceted anticancer effects of apple extracts and triterpenic compounds, however little attention has been paid to their combination with chemotherapy drugs in multidrug-resistant cancers. During the research, we found that cell lines HT-29 and U-87 were sensitive to DOX when treated with the majority of apple extract’s triterpenic complexes and individual triterpenic acids, resulting in a decrease in the viability of cancer cells.

### 3.2. Impact of Apple Extracts and Triterpenic Acids on DOX in HT-29 and U-87 Spheroid Growth (3D Cultures)

Most researchers have invoked 2D culture models, which have shown restricted opportunities in the translational to clinical practices for their deficiencies in simulating the complexity of the tumor microenvironment [34]. The application of 3D cultures to better represent the complexity of tumor biology and the microenvironment is important for screening new potential anticancer drugs. Until now, we did not find research results on the effect of apple extract′s triterpenic complexes and individual triterpenic acids on DOX in HT-29 and U-87 cell lines by simulating the physiological conditions in 3D cultures.

**Figure 2:**
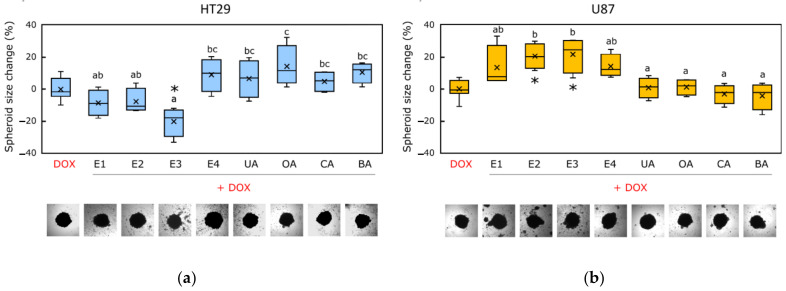
The effects of the combination of apple extract′s triterpenic complexes and individual triterpenic acids with DOX on HT-29 and U-87 spheroids’ size: (**a**) the changes in HT-29 spheroid size, and (**b**) the changes in U-87 spheroid size. Abbreviations: E1—extract of apple peel of ‘Ligol’ cultivar; E2—extract of apple peel of ‘Rubin’ cultivar; E3—extract of apple peel of ‘Auksis’ cultivar; E4—extract of the whole apple of ‘Kostele’ cultivar; UA—ursolic acid; OA—oleanolic acid; CA—corosolic acid; BA—betulinic acid. Different letters indicate statistically significant differences at p < 0.05 within the same category. Sign (*) indicates statistically significant differences at p < 0.05 within the DOX.

An analysis of 3D cell cultures showed that most combinations of apple peel extracts and DOX (marked E1 + DOX, E2 + DOX, and E3 + DOX) reduced HT-29 spheroids’ size, compared to spheroids treated with DOX alone (Figure 2a and Table S4). HT-29 spheroids’ size was most reduced by the combination of E3 extract and DOX (spheroids diameter reduced from −32.9% to −11.8%) (Figure 2a). Meanwhile, the combinations of DOX and whole apple extract (named E4 + DOX), and individual triterpenic acids (named UA + DOX, OA + DOX, CA + DOX, and BA + DOX), did not show statistically significant changes in HT-29 spheroids’ size, compared to spheroids treated with DOX alone (Figure 2a).

Unlike the previously described research results in 2D cultures, the effect of the combinations of apple extract′s triterpenic complexes and DOX on the size of U-87 spheroids was not revealed (Figure 2b). In our study, only combinations of DOX and corosolic (marked CA + DOX) and betulinic acids (marked BA + DOX) slightly decreased U-87 spheroids’ size, compared to spheroids treated with DOX alone (Figure 2b and Table S4). However, spheroids treated with E2 and E3 combinations with DOX became looser (2d), which could also indicate a toxic effect on spheroids.

**Figure 3:**
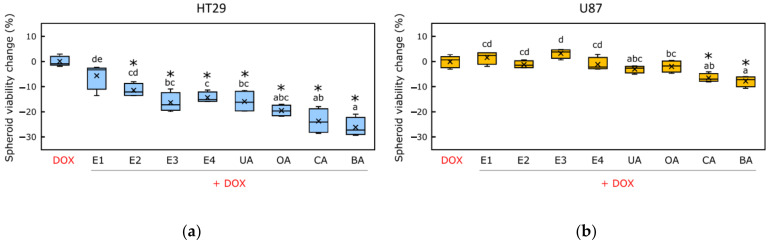
The effects of the combination of apple extract′s triterpenic complexes and individual triterpenic acids with DOX on HT-29 and U-87 spheroids’ viability: (**a**) the changes in HT-29 spheroid viability, and (**b**) the changes in U-87 spheroid viability. Abbreviations: E1—extract of apple peel of ‘Ligol’ cultivar; E2—extract of apple peel of ‘Rubin’ cultivar; E3—extract of apple peel of ‘Auksis’ cultivar; E4—extract of the whole apple of ‘Kostele’ cultivar; UA—ursolic acid; OA—oleanolic acid; CA—corosolic acid; BA—betulinic acid. Different letters indicate statistically significant differences at *p* < 0.05 within the same category. Sign (*) indicates statistically significant differences at *p* < 0.05 within the DOX.

Despite small differences in spheroid size, the MTT assay in spheroids showed more expressed differences between groups in terms of cell viability (Figure 3 and Table S5). After comparing the effects of all apple extracts and DOX combinations on spheroid viability, the combination of apple peel extract (marked E3) and DOX reduced the viability of HT-29 spheroids the most (spheroid viability reduced from –19.9% to –10.9%, compared to spheroids treated with DOX alone) (Figure 3a). From all tested apple extracts, E2 and E4 extracts combined with DOX slightly reduced the viability of U-87 spheroids (Figure 3b).

Of all tested combinations, the betulinic acid and DOX combination most efficiently reduced the viability of HT-29 spheroids (it reduced from −28.2% to −20.9%, compared to spheroids treated with DOX alone) (Figure 3a). U-87 spheroid viability was mostly reduced when incubated with combinations of DOX and corosolic or betulinic acids (Figure 3b). In the cell monolayer, the betulinic acid and DOX combination from all tested ones most efficiently reduced the viability of U-87 spheroids (spheroid viability reduced from −10.6% to −5.9%, compared to spheroids treated with DOX alone) (Figure 3b). Our results in 3D cell cultures showed that a majority of combinations of DOX and apple extracts, and individual triterpenic acids, reduced the viability of both human colon adenocarcinoma HT-29 and human glioblastoma U-87 spheroids, compared to spheroids treated with DOX alone.

Some authors reported that combinations of plant extracts and chemotherapeutic agents might act additively or synergistically. In recent decades, many studies have been conducted evaluating the effects of the combination of DOX and plant extracts on cancer cell lines. Previous studies have described more synergistic interactions between plant extracts and anticancer drugs, such as *Silybum marianum* (L.) Gaertn. Seeds’ standardized extract with DOX or paclitaxel [35], *Solanum nigrum* leaf extract and cisplatin, DOX, docetaxel, or 5-fluorouracil [36], and *Artemisia princeps* var. *orientalis* leaf extract and DOX [37].

In previous studies, we evaluated the effect of apple extract’s triterpenic and phenolic compounds on the viability of human colon adenocarcinoma HT-29 and human glioblastoma U-87 cell lines [28]. During the research, we found that the apple extracts, rich in triterpenic compound complex, had a stronger cytotoxic activity against different cell lines compared to the activity of the phenolic compounds of the apple extract [28]. Thus, to extend the previously performed studies on anticancer effects, herein we evaluated the effect of apple extract’s triterpenic complexes and individual triterpenic acids on DOX in HT-29 and U-87 cancer cells. Despite quite a large number of studies evaluating the effect of combining chemotherapeutic drugs and phenolic compounds on cancer cell lines, only fragmentary research results describing the effect of the combinations of triterpenic compounds and chemotherapeutic drugs in cancer cells are presented. Until now, we did not find results representing the effects of the combinations of triterpenic acids, or especially apple extract, with anticancer drugs in 3D cell cultures.

**Figure 4:**
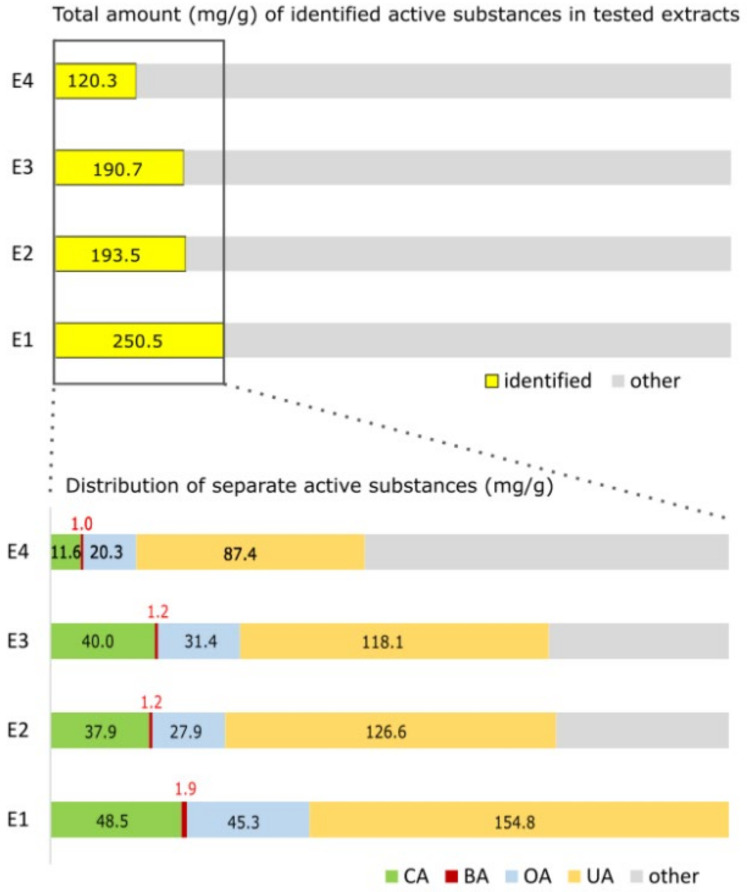
Distribution of triterpenic acids and other compounds in apple extracts. Abbreviations: E1—extract of apple peel of ‘Ligol’ cultivar; E2—extract of apple peel of ‘Rubin’ cultivar; E3—extract of apple peel of ‘Auksis’ cultivar; E4—extract of the whole apple of ‘Kostele’ cultivar; UA—ursolic acid; OA—oleanolic acid; CA—corosolic acid; BA—betulinic acid.

In our study, we found the different effects of apple extracts and individual triterpenic acids in 2D and 3D human colon adenocarcinoma and human glioblastoma spheroids’ cultures. As expected, the compounds were more active in cell monolayers than in spheroids. We determined that the human colon adenocarcinoma HT-29 cell line cultivated in a monolayer and spheroids was more sensitive to DOX when incubated with all apple extracts (E1, E2, E3, and E4) and individual triterpenic acids (UA, OA, CA, and BA) than DOX alone. Meanwhile, cell line U-87 in 2D cultures became even less sensitive to DOX when treated with all apple extracts and ursolic acid. The combination of apple extracts and DOX had no significant effect on the viability of U-87 cells in 3D cultures, compared to spheroids treated with DOX alone. After comparing the composition of the tested apple extracts, we found that the highest amounts of individual triterpenic compounds (UA, OA, CA, and BA) were found in the apple extract, marked E1 (Figure 4). However, the combination of the E1 extract and DOX had no significant effect on the viability of HT-29 and U-87 cells in 2D and 3D cultures, compared to DOX alone. Meanwhile, the combination of the E3 extract and DOX reduced the size and viability of HT-29 spheroids the most, compared to spheroids treated with DOX alone. In the E3 extract composition, as in most of the tested ones, ursolic and corosolic acids prevailed, and the lowest amount was determined for betulinic acid (Figure 4). In our study, we found that HT-29 and U-87 cells in 2D culture were the most strongly sensitive to DOX when treated with oleanolic and betulinic acids, respectively. Meanwhile, both cells in 3D culture were more sensitive to DOX when treated with betulinic acids, compared to spheroids treated with DOX alone. Vu et al. estimated that the combination of betulinic acid and DOX synergistically decreases cell viability in the MOLM-13 AML cell line but does not significantly affect the viability of U-937 cells [38].

Some authors described the effects of the combinations of individual triterpenic compounds and chemotherapeutic agents in cancer cell lines in 2D cultures. Bao et al. determined the synergistic interaction between oleanolic acid, its derivatives, and paclitaxel in breast MDA-MB-231 cancer cell lines [39]. Villar et al. established the higher antiproliferative effect of the combination of ursolic acid and DOX, compared to each agent alone. The authors showed that a combination of ursolic acid and DOX increased the effect of DOX by 2.2 to 8.7 times in synovial sarcoma cells and by 1.5 to 2.1 times in leiomyosarcoma cells [40]. Wu et al. showed that combining ursolic acid with a low dose of cisplatin significantly enhanced cytotoxicity in HepG2/DDP cells [32]. According to the literature, the effects of ursolic acid and anticancer drugs are mostly described. Perhaps the chemo-sensitive effect of DOX of apple extracts on tumor cells was determined by complexes of triterpenic acid, or maybe other not identified biologically active compounds.

We hypothesize that possibly, different apple extract concentrations of individual triterpenic acid induce different mechanisms of action in HT-29 and U-87 cell lines. The success of the combinations of triterpenic compounds and chemotherapeutic agents on cancer cells may be accompanied by various mechanisms of action and depend on the selected cancer cell types [39,41]. Our study used a chemotherapeutic agent, DOX, a topoisomerase-II inhibitor [10,38]. Meanwhile, triterpenic acids have broad-spectrum cytotoxicity effects in various cancer cells [42,43,44,45]. According to scientific literature, ursolic acid may induce cell cycle arrest and apoptosis, such as inhibition of DNA replication [46], enhancement of intracellular amounts of reactive oxygen species [47], inhibition of nuclear factor-kappa-B activation [48,49], and a reduction of anti-apoptotic proteins (such as Bcl-2, Bcl-xl, and survivin) and caspase activation [50,51,52]. Most studies have demonstrated that betulinic acid induces cancer cell death through apoptotic death, specifically by provoking the intrinsic (mitochondrial) pathway [53,54]. Some authors reported that betulinic acid in different cell types regulates Bcl-2 family proteins’ activity [55,56,57]. Oleanolic acid and its derivatives cause apoptosis and autophagy of cancer cells via inhibition of topoisomerase in cancer cells [58,59]. Triterpenic compounds may reduce the adverse effects of chemotherapeutics, for example, DOX. Previous studies reported that oleanolic acid has cardioprotective activities, and may improve hemodynamic, biochemical, and histopathological changes in DOX-induced myocardial toxicity [9]. In combination with DOX, oleanolic acid may normalize the arterial pressures, enhance the mitochondrial antioxidant mechanism, and improve left ventricular and diastolic function by increasing the inotropic and lusitropic effects of the heart [9].

Triterpenic compounds not only demonstrated a relevant apoptotic effect on cancer cells but also sensitized them to chemotherapeutic drugs [40]. Blanco et al. determined that a combination of betulinic acid and 5-fluorouracil reverted apoptosis induction in the 5-fluorouracil-resistant cells [19]. A parallel reversion activity was found with a combination of betulinic acid and oxaliplatin in oxaliplatin-resistant cells [60]. Some authors described that oleanolic acid is an inhibitor of efflux transporters, including P-glycoprotein, and in combination with paclitaxel may enhance the intracellular concentration of chemotherapeutic drugs [61]. According to previous studies, oleanolic and maslinic acids might induce apoptosis in sarcoma SW982 and SK-UT-1 cells and sensitize them to DOX [62]. In contrast, it was found that ursolic acid did not alter the uptake of DOX or increase the intracellular DOX concentration in soft-tissue sarcoma cells [40].

In some incidences, it is possible to bypass gained chemoresistance by combination therapy of anticancer agents with chemosensitizers such as triterpenic compounds. The published scientific results confirm that the combinations of individual triterpenic compounds with a chemotherapeutic drug can increase its sensitivity to cancer cells and increase cytotoxic activity. As a result, the desired effect can be achieved with a lower dose of the drug, and combined chemotherapy can reduce the consequences of side effects.

## 4. Conclusions

We evaluated that human colon adenocarcinoma HT-29 and human glioblastoma U-87 cells were sensitive to DOX when treated with apple extracts and individual triterpenic acids in 2D and 3D cultures. We determined that the HT-29 cell line was most strongly sensitive to DOX when treated with all tested apple extracts and triterpenic acids, compared to U-87 cells. HT-29 cells were most strongly sensitive to DOX when treated with 5 µM oleanolic acid. On the contrary, U-87 cells were most strongly sensitive to DOX when treated with 2 µM betulinic acid. The 3D cell cultures study showed that a combination of betulinic acid and DOX most efficiently reduced the viability of HT-29 and U-87 spheroids, compared to spheroids treated with DOX alone. Our study in 2D and 3D cultures showed that combining natural-origin apple extracts or individual triterpenic acids with DOX may sensitize it and increase the cytotoxicity effects in HT-29 and U-87 cell lines. The obtained results are important in combination chemotherapy and may help to decrease the effective dose of anticancer agents, overcome the resistance, and diminish possible side effects.

## Data Availability

All datasets generated for this study are included in the article.

## Supplementary Materials

The following supporting information can be downloaded at: https://www.mdpi.com/article/10.3390/pharmaceutics14122593/s1, Table S1: The calculated EC_50_ values (nM) of DOX and apple extracts on monolayer HT-29 and U-87 cells. Table S2: The calculated EC_50_ values (nM) of DOX and individual triterpenic compounds on monolayer HT-29 and U-87 cells. Table S3: Change of EC_50_ values determined by evaluating the effects of apple extracts, individual triterpenic compounds and DOX on monolayer HT-29 and U-87 cells. Table S4: Change of EC_50_ values determined by evaluating the effects of apple extracts, individual triterpenic compounds, and DOX on HT-29 and U-87 spheroids’ size. Table S5: Change of EC_50_ values determined by evaluating the effects of apple extracts, individual triterpenic compounds, and DOX on HT-29 and U-87 spheroids’ viability.
