# Supplementary material for: The Chemo-Sensitizing Effect of Doxorubicin of Apple Extract-Enriched Triterpenic Complex on Human Colon Adenocarcinoma and Human Glioblastoma Cell Lines
Source: Pharmaceutics. 2022 Nov 24;14(12):2593. doi: 10.3390/pharmaceutics14122593 (PMC9781225; doi:10.3390/pharmaceutics14122593)
Supplement: Supplementary file 1 [file pharmaceutics-14-02593-s001.zip › pharmaceutics-2021412-supplementary.pdf]

Article

The Chemosensitizing Effect of Doxorubicin of Apple Extract-Enriched Triterpenic Complex on Human Colon Adenocarcinoma and Human Glioblastoma Cell Lines

Aurita Braciuliene [1,*], Valdimaras Janulis [1] and Vilma Petrikaite [2]

[1] Department of Pharmacognosy, Lithuanian University of Health Sciences, Sukileliu av. 13, LT-50162 Kaunas, Lithuania;
[2] Laboratory of Drug Targets Histopathology, Institute of Cardiology, Lithuanian University of Health Sciences, Sukileliu av. 13, LT-50162 Kaunas, Lithuania;
* Correspondence: Aurita.Braciuliene@lsmu.lt (A.B.); Tel.: +37-037-621-56190

Supplementary Material:

Table S1. The calculated EC_{50} values (nM) of DOX and apple extracts on monolayer HT-29 and U-87 cells.

Sample	HT-29			U-87		
	1	2	3	1	2	3
DOX	21	22	50	16	33	32
E1+DOX	13	12	27	20	36	36
E2+DOX	8	12	26	23	40	44
E3+DOX	10	12	25	17	35	35
E4+DOX	10	10	24	18	34	36

Abbreviations: E1 – extract of apple peel of 'Ligol' cultivar; E2 − extract of apple peel of 'Rubin' cultivar; E3 − extract of apple peel of 'Auksis' cultivar; E4 − extract of the whole apple of 'Kostele' cultivar; UA – ursolic acid; OA – oleanolic acid; CA – corosolic acid; BA – betulinic acid; DOX– doxorubicin.

Table S2. The calculated EC_{50} values (nM) of DOX and individual triterpenic compounds on monolayer HT-29 and U-87 cells.

Sample	HT-29			U-87		
	1	2	3	1	2	3
DOX	40	50	50	35	20	32
UA+DOX	14	24	24	38	22	24
OA+DOX	14	20	14	30	17	26
CA+DOX	25	22	32	26	15	20
BA+DOX	20	21	30	18	12	17

Abbreviations: E1 – extract of apple peel of 'Ligol' cultivar; E2 − extract of apple peel of 'Rubin' cultivar; E3 − extract of apple peel of 'Auksis' cultivar; E4 − extract of the whole apple of 'Kostele' cultivar; UA – ursolic acid; OA – oleanolic acid; CA – corosolic acid; BA – betulinic acid; DOX– doxorubicin.

Table S3. Change of EC_{50} values determined by evaluating the effects of apple extracts, individual triterpenic compounds and DOX on monolayer HT-29 and U-87 cells.

Sample	HT-29		U-87	
	Average, %	SD	Average, %	SD
UA+DOX	–61,9	8,8	8,3	1,9
OA+DOX	–64,6	4,4	–16,0	2,4
CA+DOX	–43,9	10,7	–29,4	7,0
BA+DOX	–49,3	9,0	–45,1	4,5
E1+DOX	–43,2	2,2	15,5	8,4
E2+DOX	–51,8	8,9	34,2	1,7
E3+DOX	–49,3	3,5	7,2	0,4
E4+DOX	–53,0	1,4	9,3	5,5

Abbreviations: E1 – extract of apple peel of 'Ligol' cultivar; E2 − extract of apple peel of 'Rubin' cultivar; E3 − extract of apple peel of 'Auksis' cultivar; E4 − extract of the whole apple of 'Kostele' cultivar; UA – ursolic acid; OA – oleanolic acid; CA – corosolic acid; BA – betulinic acid; DOX– doxorubicin.

Table S4. Change of EC_{50} values determined by evaluating the effects of apple extracts, individual triterpenic compounds and DOX on HT-29 and U-87 spheroids size.

Sample	HT-29		U-87	
	Average, %	SD	Average, %	SD
DOX	0,0	7,0	0,0	5,8
UA+DOX,	6,6	11,7	0,8	6,4
OA+DOX	14,3	13,0	1,2	5,2
CA+DOX	4,9	6,5	–3,1	6,1
BA+DOX	10,6	6,4	–4,3	8,4
E1+DOX	−8,5	8,1	13,4	13,3
E2+DOX	−7,6	7,8	20,5	7,8
E3+DOX	−20,1	9,1	21,6	11,1
E4+DOX	9,1	10,4	14,1	7,4

Abbreviations: E1 – extract of apple peel of 'Ligol' cultivar; E2 − extract of apple peel of 'Rubin' cultivar; E3 − extract of apple peel of 'Auksis' cultivar; E4 − extract of the whole apple of 'Kostele' cultivar; UA – ursolic acid; OA – oleanolic acid; CA – corosolic acid; BA – betulinic acid; DOX– doxorubicin.

Table S5. Change of EC_{50} values determined by evaluating the effects of apple extracts, individual triterpenic compounds and DOX on HT-29 and U-87 spheroids viability.

Sample	HT-29		U-87	
	Average, %	SD	Average, %	SD
DOX	0,0	1,9	0,0	2,3
UA+DOX	−15,9	4,2	−2,9	1,4
OA+DOX	−19,5	2,2	−1,9	2,3
CA+DOX	−23,6	4,9	−6,5	1,7
BA+DOX	−26,1	3,7	−7,7	2,1
E1-DOX	−5,7	5,3	0,9	2,5
E2-DOX	−11,5	2,6	−1,1	1,5
E3-DOX	−16,3	3,8	3,3	1,8
E4-DOX	−14,4	2,1	−1,1	2,6

Abbreviations: E1 – extract of apple peel of 'Ligol' cultivar; E2 − extract of apple peel of 'Rubin' cultivar; E3 − extract of apple peel of 'Auksis' cultivar; E4 − extract of the whole apple of 'Kostele' cultivar; UA – ursolic acid; OA – oleanolic acid; CA – corosolic acid; BA – betulinic acid; DOX– doxorubicin.
